# Upper lumbar disc herniation presenting as chronic right lower abdominal pain in a patient with multilevel lumbar degeneration: a case report

**DOI:** 10.3389/fsurg.2026.1864618

**Published:** 2026-06-19

**Authors:** Mingyang Wei, Jiantao Shi, Yi Dai, Lei Jiao, Wei Li, Jianhua Sun

**Affiliations:** First Affiliated Hospital of Shihezi University, Shihezi, Xinjiang Uygur Autonomous Region, China

**Keywords:** case report, multilevel lumbar degeneration, radicular referred pain, right lower abdominal pain, selective nerve root block, staged treatment, upper lumbar disc herniation

## Abstract

Upper lumbar disc herniation (ULDH) is relatively uncommon and may present with atypical symptoms, including lower abdominal pain, which can complicate clinical diagnosis. The diagnostic challenge is even greater when multilevel lumbar degeneration is present and the symptomatic level is unclear. We report the case of a 65-year-old woman with right lower abdominal pain for more than 5 years. The pain worsened during the 3 months before admission and was accompanied by low back pain and radiating pain in the left lower limb. Repeated abdominal evaluations, including abdominal ultrasonography and gynecological and urological assessments, did not reveal any abnormality sufficient to explain her symptoms. Lumbar magnetic resonance imaging showed multilevel degenerative changes, including L2–3 disc herniation and L4–5 spinal canal stenosis with degenerative spondylolisthesis. Because the imaging findings did not fully correspond to the clinical presentation, fluoroscopy-guided right L2–3 selective nerve root block (SNRB) was performed to help identify the responsible level. After the block, the visual analogue scale (VAS) score for right lower abdominal pain decreased from 7 to 3, although the pain returned to the pre-block level 2 days later. The patient subsequently underwent right L2–3 transforaminal endoscopic lumbar discectomy (TELD), after which the VAS score for right lower abdominal pain decreased further to 2. Persistent low back pain and left lower-limb radiating pain were then treated with L4–5 posterior lumbar interbody fusion (PLIF), and the VAS score for left leg pain decreased to 2. This case suggests that radicular referred pain caused by ULDH should be considered in patients with long-standing lower abdominal pain and repeatedly negative abdominal evaluations, particularly when spinal symptoms coexist. In patients with multilevel degeneration and discordant clinical and imaging findings, SNRB may help localize the symptomatic level and support a staged, symptom-oriented surgical strategy.

## Introduction

Upper lumbar disc herniation (ULDH) is uncommon, accounting for approximately 1%–11% of all lumbar disc herniations (1, 2). Its clinical presentation is often nonspecific. Patients may present with pain in the groin, lower abdomen, or anteromedial thigh rather than with typical dermatomal radiating pain (3, 4). As a result, gastrointestinal, urological, or gynecological disorders are often investigated first, and a spinal etiology may be considered only after a delay. Previous studies and case reports have shown that thoracolumbar junction or upper lumbar lesions can present with atypical symptoms such as abdominal pain, groin pain, scrotal pain, and abdominal pseudohernia (5–8). However, most published reports have focused primarily on recognizing these atypical symptoms. In patients with multilevel lumbar degeneration, a more practical clinical problem is determining which of several radiographic abnormalities is truly responsible for the patient's symptoms.

Selective nerve root block (SNRB) involves image-guided injection of a local anesthetic around a target nerve root, with subsequent assessment of symptom change to help identify the symptomatic root. Although its diagnostic accuracy has limitations, SNRB can provide clinically useful information when symptoms are atypical or when imaging shows multilevel abnormalities (9–11). Once a likely responsible level has been identified, targeted minimally invasive decompression may relieve symptoms and further clarify the relationship between the clinical presentation and imaging findings. We report a case of chronic right lower abdominal pain in a patient with multilevel lumbar degeneration. By integrating the pain distribution, repeated negative abdominal evaluations, SNRB findings, and outcomes after staged surgical treatment, the most likely source of the patient's symptoms was identified. The case also shows how symptom separation, targeted block, and staged surgery can be used when abdominal pain and lower-limb radicular symptoms appear to arise from different lumbar levels.

## Case presentation

A 65-year-old woman presented with recurrent right lower abdominal pain that had persisted for more than 5 years. During the 3 months before admission, the pain worsened and was accompanied by low back pain and radiating pain in the left lower limb. She had previously undergone repeated abdominal evaluations at other hospitals, including abdominal ultrasonography and gynecological and urological assessments, but no definitive cause had been identified. Abdominal examination showed no signs of peritoneal irritation; however, there was marked localized tenderness in the right lower abdomen. The patient described the pain as beginning in the right lower back and radiating along the flank toward the right lower abdomen (Figure 1). Neurological examination showed a positive straight-leg-raising test on the left at 35 degrees, a suspected positive femoral nerve stretch test on the left, partial sensory reduction in the left lower limb, and largely preserved muscle strength.

**Figure 1:**
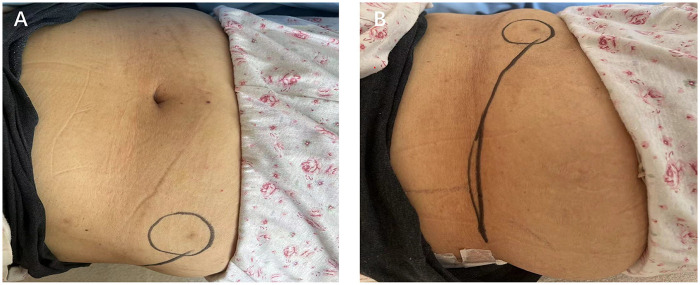
Distribution of the patient's pain. **(A)** Localized point of maximal pain in the right lower abdomen. **(B)** Patient-reported pain trajectory extending from the right lower back/flank toward the right lower abdomen.

During hospitalization, gastrointestinal surgical consultation was obtained because right lower abdominal pain was the dominant complaint. Routine laboratory tests did not reveal a clear abnormality. The consultation record documented no obvious rebound tenderness and recommended further colonoscopic and abdominal/pelvic computed tomography (CT) evaluation; abdominal and pelvic computed tomography showed no definite abnormality sufficient to explain the chronic right lower abdominal pain (Figure 2). No definite gastrointestinal, urological, or gynecological disorder was identified in the available clinical records. Lumbar magnetic resonance imaging (MRI) demonstrated multilevel degenerative changes, including L2–3 disc herniation, L4–5 spinal canal stenosis, and degenerative spondylolisthesis (Figure 3). Given the repeatedly negative abdominal evaluations, the characteristic pain distribution, and the accompanying neurological signs, multidisciplinary discussions during outpatient and inpatient care suggested that an intra-abdominal visceral cause of the long-standing right lower abdominal pain was unlikely and that radicular referred pain should be considered. The L4–5 lesion could reasonably explain the low back pain and left lower-limb radiating pain, but it did not adequately account for the long-standing, predominant right lower abdominal pain. In contrast, the L2–3 lesion was more consistent with the pain distribution and the overall clinical course. Therefore, this level was selected for further functional assessment.

**Figure 2:**
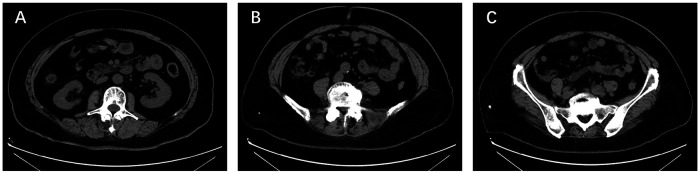
Abdominal and pelvic computed tomography images. **(A–C)** Axial CT images showing no definite lesion sufficient to explain the patient's chronic right lower abdominal pain.

**Figure 3:**
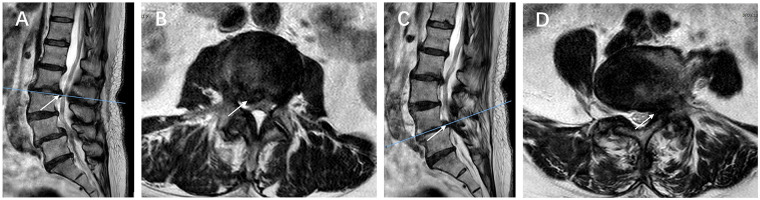
Preoperative lumbar MRI. **(A)** Sagittal T2-weighted image showing disc herniation at L2–3 with compression of the thecal sac (arrow). **(B)** Axial T2-weighted image at L2–3 showing central-to-right paracentral disc herniation and associated spinal canal narrowing (arrow). **(C)** Sagittal T2-weighted image showing degenerative changes at L4–5 with spinal canal stenosis and degenerative spondylolisthesis (arrow). **(D)** Axial T2-weighted image at L4–5 showing left-sided lateral recess/foraminal stenosis with compression of the corresponding nerve root (arrow).

To further identify the level associated with the right lower abdominal pain, fluoroscopy-guided right L2–3 SNRB was performed. Under local anesthesia, fluoroscopy was used to confirm the needle position at the right L2–3 level and a mixture of 3 mL lidocaine (20 mg/mL) and 1 mL triamcinolone acetonide (40 mg/mL) was then injected. The visual analogue scale (VAS) score for right lower abdominal pain was assessed 30 min after the block and decreased from 7 to 3, suggesting that the L2–3 lesion was involved in the abdominal pain. The analgesic effect was temporary, and the pain returned to the pre-block level 2 days later. Based on the response to the block and the imaging findings, the patient underwent right L2–3 TELD under local anesthesia 2 days later. Foraminal plasty was performed, the herniated disc material was removed, and adequate nerve root decompression was achieved. After surgery, the VAS score for right lower abdominal pain decreased to 2, further supporting L2–3 as the level most closely related to this symptom.

After the first-stage procedure, the patient's right lower abdominal pain improved markedly, but she continued to experience persistent low back pain and radiating pain in the left lower limb, which affected her daily activities. Preoperative imaging demonstrated L4–5 spinal canal and foraminal stenosis with compression of the corresponding nerve root, and dynamic flexion-extension radiographs suggested segmental instability at L4–5. Therefore, L4–5 posterior lumbar interbody fusion (PLIF) was performed as a second-stage operation to decompress the affected nerve root and restore segmental stability. After PLIF, the VAS score for left leg pain decreased to 2. Lumbar magnetic resonance imaging at the 3-month follow-up showed marked relief of neural compression at L2–3 and substantial improvement in L4–5 spinal canal stenosis (Figure 4). During 12 months of follow-up, the abdominal pain did not recur, and the left lower-limb symptoms remained improved. The clinical timeline is summarized in Table 1.

**Figure 4:**
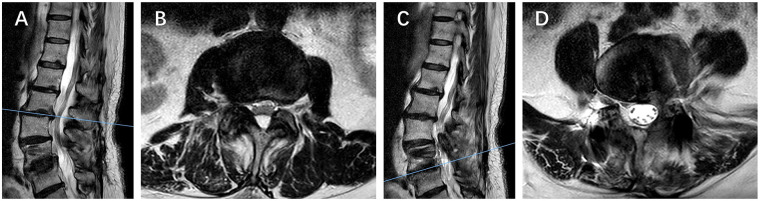
Postoperative lumbar MRI obtained 3 months after the second-stage surgery. **(A)** Sagittal T2-weighted image showing postoperative changes at L2–3 after TELD, with reduction of the previously herniated disc material and decreased thecal sac compression. **(B)** Axial T2-weighted image at L2–3 showing postoperative decompression with enlargement of the spinal canal compared with the preoperative image. **(C)** Sagittal T2-weighted image showing postoperative changes at L4–5 after PLIF, with improvement in the previously noted degenerative spondylolisthesis and spinal canal stenosis. **(D)** Axial T2-weighted image at L4–5 showing postoperative changes with reduced left-sided lateral recess/foraminal stenosis compared with the preoperative image.

**Table 1:** Clinical timeline.

Time	Clinical event
>5 years before admission	Recurrent right lower abdominal pain; repeated abdominal workup was unrevealing.
3 months before admission	Worsening abdominal pain with low back pain and left leg radiating pain.
Preoperative evaluation	Abdominal workup was negative; MRI showed L2–3 disc herniation and L4–5 stenosis/spondylolisthesis.
Diagnostic procedure	Right L2–3 SNRB; abdominal pain VAS score decreased from 7 to 3.
First-stage surgery	Right L2–3 TELD; abdominal pain VAS score decreased to 2.
Second-stage surgery	L4–5 PLIF; left leg pain VAS score decreased to 2.
3-month follow-up	MRI showed decompression at L2–3 and improvement of L4–5 stenosis.
12-month follow-up	Abdominal pain did not recur; left leg symptoms improved.

## Discussion

Lower abdominal pain caused by upper lumbar pathology is uncommon and may be easily overlooked, particularly when abdominal symptoms dominate the clinical picture and typical radicular complaints are absent or subtle. In such circumstances, the diagnostic pathway usually begins with gastrointestinal, urological, or gynecological evaluation, and a spinal origin may be considered only later. In the three cases reported by Babaei et al. (7), patients were initially evaluated for hernia, appendicitis, or peripheral neuralgia; after laboratory tests and abdominal imaging failed to reveal a clear abnormality, spinal MRI ultimately identified thoracolumbar or upper lumbar foraminal lesions. Similar to those cases, our patient had long-standing right lower abdominal pain as the main complaint, and repeated abdominal evaluations did not reveal an intra-abdominal lesion sufficient to explain the pain. Radicular referred pain should therefore be considered earlier in similar patients, especially when repeated abdominal evaluations are unrevealing.

Anatomically, sensory innervation of the lower abdominal wall and groin is closely related to the thoracolumbar and upper lumbar nerve roots. Previous studies have noted that the iliohypogastric and ilioinguinal nerves arise mainly from the L1–2 roots, whereas abdominal wall sensory innervation also involves the T10–L1 segments (7, 12, 13). Accordingly, thoracolumbar junction or upper lumbar disc and foraminal lesions may present as lower abdominal or groin pain rather than typical lower-limb radiating pain. In the present case, the L2–3 disc herniation was predominantly located in the right paracentral/lateral recess region, causing compression of the thecal sac and narrowing of the right lateral recess. Based on this anatomical location, the traversing right L3 nerve root was considered the most likely nerve root involved. Although the L3 dermatome does not classically correspond to the lower abdominal wall, pain caused by lumbar pathology does not always conform strictly to classic dermatomal maps. Oikawa et al. (6) suggested that discogenic pain may be transmitted through sympathetic-related pathways and multisegmental afferent mechanisms, particularly involving the L2 dorsal root ganglion, thereby producing groin or lower abdominal pain. Tang et al. (5) further proposed the concept of discogenic visceral pain, indicating that pain arising from spinal lesions can clinically mimic visceral abdominal pain. In this patient, the right-sided L2–3 lesion, the pain trajectory from the right lower back/flank toward the right lower abdomen, the marked but temporary relief after right L2–3 SNRB, and the improvement after L2–3 decompression strongly suggested that the right lower abdominal pain was most likely associated with upper lumbar nerve root irritation at the L2–3 level. However, because this is a single case and the pain distribution was atypical, this relationship should be interpreted as clinically suggestive rather than definitive.

The main value of this case lies not only in the atypical symptom pattern but also in demonstrating how to identify the symptomatic level in the setting of multilevel lumbar degeneration. Older patients often have concurrent multilevel disc degeneration, spinal canal stenosis, and segmental instability, and the most conspicuous abnormality on imaging is not necessarily the source of the main symptom. In the present case, the L4–5 lesion reasonably explained the left lower-limb radiating pain but could not explain the long-standing right lower abdominal pain that dominated the clinical presentation. By contrast, the L2–3 lesion was more consistent with the pain distribution and symptom evolution. This distinction had direct therapeutic implications: if treatment had targeted only the most obvious degenerative level on imaging, the symptom most important to the patient might have persisted.

In this context, SNRB provided important diagnostic support. Yeom et al. (14) reported that SNRB has value in localizing the symptomatic nerve root, whereas the systematic review by Beynon et al. (15) noted that its specificity and overall diagnostic reliability are not constant; therefore, SNRB should not be regarded as an independent gold standard. For this reason, SNRB findings should be interpreted together with the history, physical examination, imaging findings, and clinical outcome. In this patient, the marked but temporary improvement in lower abdominal pain after right L2–3 block supported L2–3 as the most likely source of the abdominal component. However, this response was not treated as decisive evidence in isolation. Instead, it was interpreted alongside the repeatedly negative abdominal evaluations, the pain distribution, the imaging findings, and the postoperative course. In patients with multilevel degenerative disease, this type of integrated judgment is often more useful for surgical decision-making than reliance on imaging alone.

Another important feature of this case is the clinical value of staged treatment. After the right L2–3 level was identified as the suspected symptomatic level for the right lower abdominal pain, first-stage targeted decompression with TELD was performed. The aim was not to treat every radiographic abnormality at once, but to relieve the dominant symptom with minimal surgical trauma and further test the presumed symptomatic level. At the same time, the patient's low back pain and left lower-limb radiating pain did not fully resolve after L2–3 surgery. These symptoms were consistent with the L4–5 lesion, where imaging showed degenerative stenosis/spondylolisthesis and segmental instability with compression of the left-sided neural structures. Subsequent improvement after L4–5 PLIF suggested that different symptom components arose from different spinal levels. In this sense, staged treatment had both therapeutic and explanatory value, helping to clarify the respective contributions of coexisting lesions within the overall symptom profile.

## Limitations

As a single case report, this study has several limitations. First, the causal relationship between the L2–3 lesion and chronic right lower abdominal pain cannot be definitively proven from a single patient. Although the convergence of the pain trajectory, repeatedly negative abdominal evaluations, temporary relief after right L2–3 selective nerve root block, and postoperative improvement after L2–3 decompression strongly suggested that the abdominal pain was most likely associated with the right-sided L2–3 lesion, this interpretation should be regarded as clinically suggestive rather than definitive. Second, SNRB was an important adjunct for identifying the suspected symptomatic level, but it should not be considered a stand-alone diagnostic criterion. Possible spread of the injected anesthetic and corticosteroid to adjacent structures may reduce its specificity; therefore, the block result should be interpreted in combination with clinical symptoms, physical examination, imaging findings, and surgical outcome. Finally, because this report describes an uncommon presentation in one patient, further studies with larger samples and standardized diagnostic protocols are needed to better define the relationship between upper lumbar disc disease and atypical lower abdominal pain.

## Conclusions

In patients with long-standing unexplained lower abdominal pain, especially when abdominal evaluations are repeatedly negative and spinal symptoms coexist, radicular referred pain caused by ULDH should be considered. When multilevel lumbar degeneration is present and symptoms do not fully match imaging findings, SNRB can serve as an important adjunct for identifying the symptomatic level. This case highlights the importance of symptom stratification and responsible-level determination in complex multilevel lumbar degenerative disease. A staged, symptom-oriented treatment strategy may help distinguish the sources of different symptoms and provide a useful diagnostic and therapeutic reference for similar cases of atypical lower abdominal pain.

## Data Availability

The original contributions presented in the study are included in the article/Supplementary Material, further inquiries can be directed to the corresponding author.
